# Boiled Sausage Sign: A Novel MRI Marker for Identifying Subacute Secondary Hydrocephalus in the Elderly

**DOI:** 10.7759/cureus.80587

**Published:** 2025-03-14

**Authors:** Miharuka Yokosaki, Shuichiro Neshige, Hirofumi Maruyama

**Affiliations:** 1 Department of Clinical Neuroscience and Therapeutics, Hiroshima University, Hiroshima, JPN

**Keywords:** boiled sausage sign, brain atrophy, meningeal carcinomatosis, neuro-emergency, subacute secondary hydrocephalus

## Abstract

Ventricular enlargement is commonly caused by brain atrophy or hydrocephalus; however, distinguishing between these conditions in elderly patients is often challenging. This case report describes a patient in whom secondary hydrocephalus was suspected based on characteristic findings of ventricular enlargement, leading to the final diagnosis. A 75-year-old man with nasal cavity malignant melanoma, treated with nivolumab for six months, presented with three weeks of progressive lethargy and anorexia and subsequent mild confusion. Although MRI-negative encephalitis related to nivolumab was initially suspected, mild ventricular enlargement with a uniformly dilated anterior horn shape resembling a "boiled sausage" was noted. Subsequent gadolinium-enhanced MRI demonstrated diffuse dural thickening contiguous with the nasal cavity and meningeal enhancement, leading to the final diagnosis of intracranial invasion of malignant melanoma with secondary hydrocephalus due to meningeal carcinomatosis. Subacute hydrocephalus manifests as a uniform ventricular enlargement on MRI, a finding we propose as the novel "boiled sausage sign." This sign may help distinguish secondary hydrocephalus from physiologic atrophy, even when the Evans index remains within the normal range.

## Introduction

Ventricular enlargement is commonly observed in elderly patients. While this condition is often attributed to brain atrophy [1,2], it can also be seen in hydrocephalus, such as idiopathic normal pressure hydrocephalus (iNPH) or secondary hydrocephalus due to a particular acute brain disease [3,4]. However, distinguishing between these conditions in elderly patients is often challenging and requires specific brain imaging findings that can differentiate them [5,6]. In the present case, we found that the characteristic shape of mild ventricular enlargement with a uniformly dilated anterior horn, resembling a "boiled sausage," might be associated with subacute secondary hydrocephalus rather than brain atrophy. We operationally termed this finding the "boiled sausage sign." Since the subsequent evaluation led to a diagnosis of secondary hydrocephalus due to carcinomatous meningitis, we speculated that the "boiled sausage sign" might be useful in identifying the etiology of ventricular enlargement in the elderly. When this sign is present, a thorough evaluation for possible causes other than brain atrophy may facilitate an accurate diagnosis.

## Case presentation

A 75-year-old man presented with progressive lethargy and anorexia for three weeks, followed by gait instability and urinary incontinence, eventually leading to confusion. He had a history of malignant melanoma in the nasal cavity and had been receiving immunotherapy with nivolumab for six months. Prior to onset, there was no clinical history of dementia or related disorders, including iNPH. Vital signs were within the normal range. Neurological examination revealed drowsiness, with a Glasgow Coma Scale score of 10 (E2V4M4), and positive signs of meningeal irritation. Cerebrospinal fluid (CSF) analysis revealed clear, watery fluid, an initial CSF pressure of 18.5 mmH₂O, a CSF cell count of 47/μL (pleocytosis 82%), and a CSF protein level of 151 mg/dL. However, there was no evidence of infection in CSF analysis or culture testing (Table 1).

**Table 1:** Laboratory investigations CSF: cerebrospinal fluid

Test (CSF)	Observed value	Reference range
Appearance	Clear	Clear
Opening pressure	18.5 cmH₂O	7-18 cmH₂O
Cell count	47/µL	0-5/µL
Mononuclear cell	39/µL	0-5/µL
Polymorphonuclear cell	8/µL	0-5/µL
Protein	151 mg/dL	15-45 mg/dL
Glucose	50 mg/dL	50-75 mg/dL
Albmin	66.6 mg/dL	9-30 mg/dL
IgG	26.1 mg/dL	0.5-4 mg/dL
IgG index	0.76	< 0.7
Na	142 mmol/L	135-150 mmol/L
Cl	120 mmol/L	118-128 mmol/L
Oligoclonal bands	Negative	Negative
Gram stain	No organisms seen	
Culture	No growth	
Cytology	Negative	Negative

**Figure 1:**
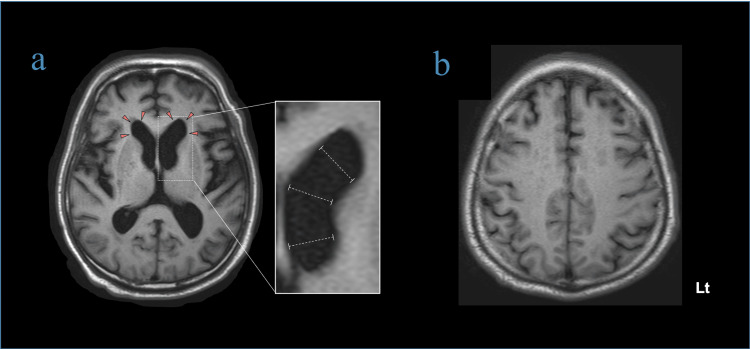
Axial T1-weighted brain MRI a. Mild ventricular enlargement is observed bilaterally at the anterior horn (red arrowheads). Diameters at the distal, mid, and proximal portions are approximately equal, forming a pattern termed the “boiled sausage sign.” b. Cortical atrophy is not observed. Narrowing of the cerebral sulci in the high convexity region is not observed.

The initial brain MRI was performed on the same day as the lumbar puncture and showed no remarkable findings. Thus, given his history of nivolumab treatment, MRI-negative immune-related adverse events (irAEs) encephalitis was initially suspected [7], and steroid therapy was considered. However, mild ventricular enlargement resembling a "boiled sausage" was noted without cortical atrophy (Figure 1).

**Figure 2:**
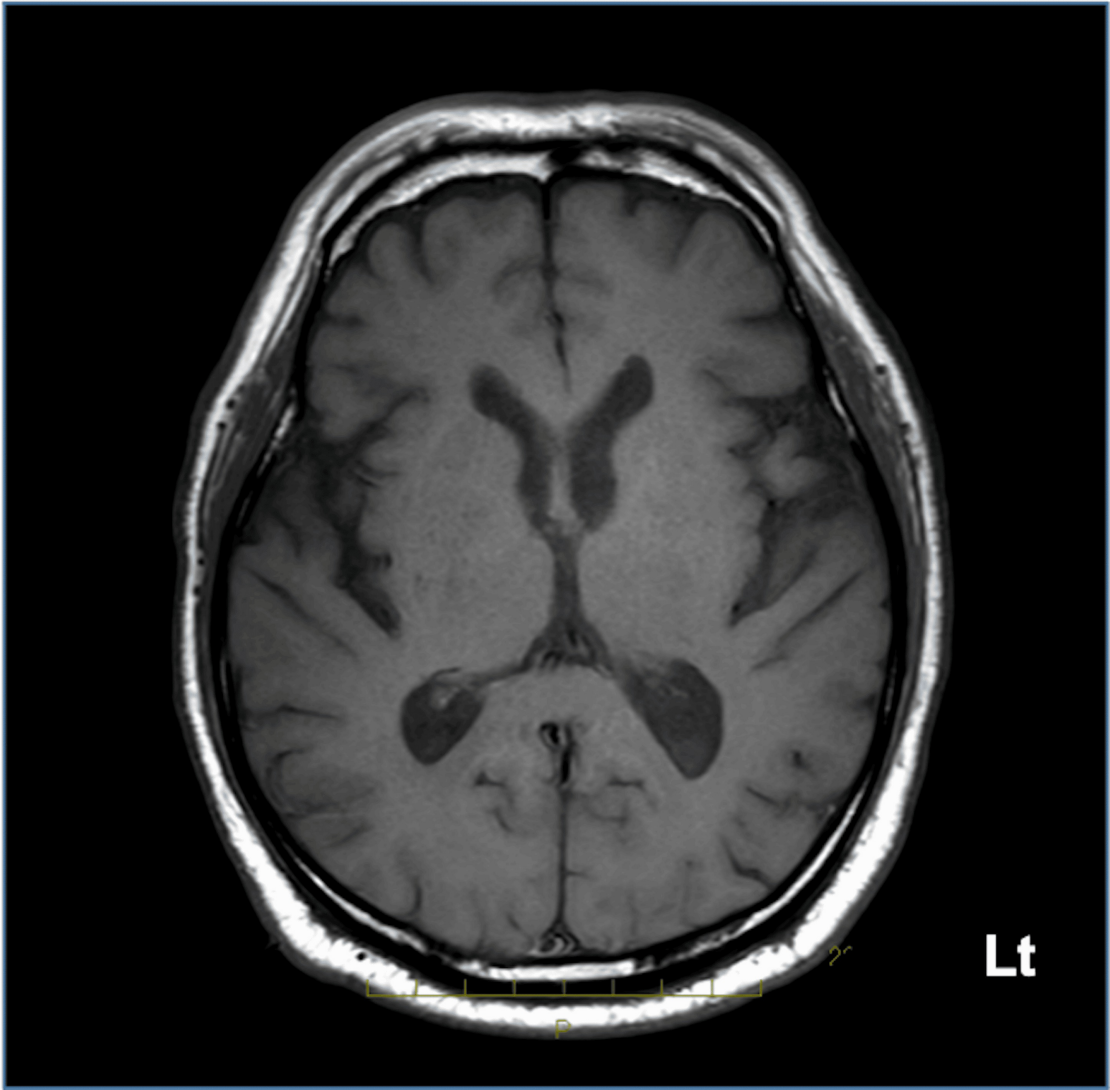
T1-weighted brain MRI of the same cross-section from one year ago A ventricular enlargement is not observed.

Additionally, compared with the previous brain MRI, ventricular enlargement had progressed (Figure 2).

**Figure 3:**
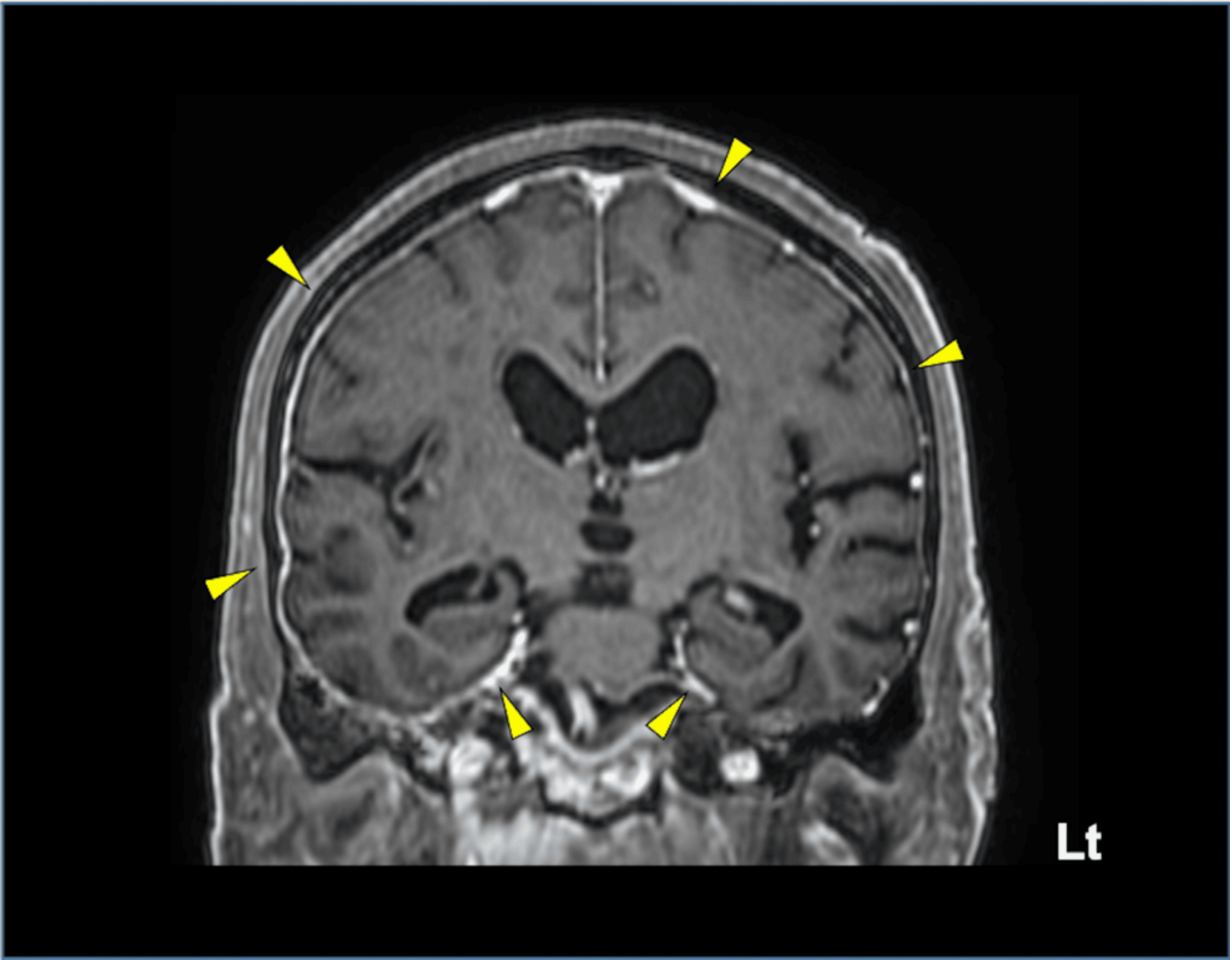
Gadolinium-enhanced T1-weighted brain MRI Diffuse dural thickening with enhancement evident bilaterally (yellow arrowheads).

Therefore, gadolinium-enhanced MRI was performed to clarify the etiology, revealing diffuse dural thickening contiguous with the nasal cavity with meningeal enhancement. Furthermore, in the coronal view, ventricular enlargement was observed without narrowing of the cerebral sulci in the high convexity region, a characteristic feature of iNPH (Figure 3) [8].

Subsequent work-up confirmed a final diagnosis of intracranial invasion of malignant melanoma, with secondary hydrocephalus due to meningeal carcinomatosis.

## Discussion

This case highlights intracranial invasion of malignant melanoma, with secondary hydrocephalus caused by meningeal carcinomatosis. Although MRI-negative irAE encephalitis was initially suspected given his immunotherapy history and subacute clinical progression [7], a careful review of brain imaging studies identified mild ventricular enlargement, prompting further investigation with gadolinium-enhanced MRI, which ultimately confirmed the diagnosis. However, in the absence of prior MRI, early identification of subacute hydrocephalus as the cause of ventricular enlargement can be challenging, particularly in elderly patients [5,6]. In this case, the lateral ventricles displayed a characteristic shape with rounded, uniformly dilated anterior horns, resembling a "boiled sausage." We called this finding the "boiled sausage sign" as a novel imaging sign.

In iNPH, ventricular enlargement is often associated with cortical thinning and a significant surface expansion, mainly in areas located in the medial aspects of the frontal horns and the superior portion of the bilateral lateral ventricles, which are surrounded by the high convexity of the frontal and parietal regions and the medial frontal lobe [9]. Because age-related ventricular enlargement results from cortical thinning in the medial frontal horns and superior portions of the lateral ventricles, the ventricles are presumed to dilate irregularly, as is occasionally seen in iNPH. Thus, the diagnostic significance of this sign depends on the relative absence of cortical brain atrophy. We propose that this sign may assist in distinguishing subacute hydrocephalus from cerebral atrophy. Unlike atrophic ventriculomegaly, which typically presents with irregular ventricular dilation and greater expansion in the mid-portions, subacute hydrocephalus, as demonstrated by the sign, exhibits more uniform ventricular enlargement. We speculated that secondary hydrocephalus without cortical atrophy exhibits a more uniform ventricular enlargement due to even pressure exerted on the lateral ventricles. In this case, characterized by malignancy and elevated CSF cell counts without signs of inflammation or infection, the "boiled sausage sign" was observed on plain MRI. This finding prompted further investigation with gadolinium-enhanced MRI to evaluate potential causes of ventricular enlargement other than brain atrophy, ultimately leading to the accurate diagnosis of meningeal carcinomatosis. Thus, this sign may be diagnostically useful even when the Evans index remains normal (<0.3) and typical features of hydrocephalus are absent [10], as shown in our case. Therefore, when this sign is present on plain MRI, differential diagnoses other than cerebral atrophy should be considered. Further detailed evaluations, including gadolinium-enhanced MRI, may help identify underlying conditions associated with secondary hydrocephalus. However, this hypothesis remains speculative, and additional cases are necessary to validate the diagnostic utility of this imaging sign.

## Conclusions

Mild ventricular enlargement characterized by uniformly dilated anterior horns, which we propose as the "boiled sausage sign," may help differentiate secondary hydrocephalus from brain atrophy. The diagnostic value of this sign lies in recognizing ventricular enlargement without cortical atrophy, facilitating differentiation between these conditions even when the Evans index is within the normal range. Thus, the presence of this sign on MRI warrants a detailed evaluation of alternative causes other than brain atrophy, potentially leading to an accurate diagnosis.
